# Plant–Pathogen Interaction-Related MicroRNAs and Their Targets Provide Indicators of Phytoplasma Infection in *Paulownia tomentosa* × *Paulownia fortunei*

**DOI:** 10.1371/journal.pone.0140590

**Published:** 2015-10-20

**Authors:** Guoqiang Fan, Suyan Niu, Tong Xu, Minjie Deng, Zhenli Zhao, Yuanlong Wang, Lin Cao, Zhe Wang

**Affiliations:** 1 Institute of Paulownia, Henan Agricultural University, Zhengzhou, Henan, P.R. China; 2 Beijing Genomics Institute, Shenzhen, Guangdong, P.R. China; Cankiri Karatekin University, TURKEY

## Abstract

Paulownia witches’ broom (PaWB) caused by a phytoplasma, has caused extensive losses in the yields of paulownia timber and resulted in significant economic losses. However, the molecular mechanisms in Paulownia that underlie the phytoplasma stress are poorly characterized. In this study, we use an Illumina platform to sequence four small RNA libraries and four degradome sequencing libraries derived from healthy, PaWB-infected, and PaWB-infected 15 mg·L^−1^ and 30 mg·L^−1^ methyl methane sulfonate (MMS)-treated plants. In total, 125 conserved and 118 novel microRNAs (miRNAs) were identified and 33 miRNAs responsive to PaWB disease were discovered. Furthermore, 166 target genes for 18 PaWB disease-related miRNAs were obtained, and found to be involved in plant-pathogen interaction and plant hormone signal transduction metabolic pathways. Eleven miRNAs and target genes responsive to PaWB disease were examined by a quantitative real-time PCR approach. Our findings will contribute to studies on miRNAs and their targets in Paulownia, and provide new insights to further understand plant-phytoplasma interactions.

## Introduction

Phytoplasma are specialized bacteria, generally called mycoplasma-like organisms, which belong to the Mollicutes class. They live in the phloem tissue of plants and are transmitted by insect vectors [1]. It has been reported that phytoplasmas are the pathogens associated with a large number of diseases in several hundred economically important plants [2, 3], and Paulownia plants are no exception [4, 5]. Paulownia witches’ broom (PaWB) disease, caused by the phytoplasma in the subgroup 16SrI-D of aster yellows [1, 6], has resulted in extensive losses in the yields of paulownia timber, leading to significant economic losses. When Paulownia plants become infected, the phytoplasma can move systemically by the phloem sieve tube elements and mainly accumulate in developing leaves, flowers, and roots, where symptoms including yellow/purple discoloration of leaves and shoots, virescence (greening of petals), phyllody (conversion of floral organs into leaf-like structures), proliferation of shoots, witches’ broom, and stunting appear. In some cases PaWB infection causes plant death.

Many researchers have been working on how to prevent and control the damage caused by PaWB disease. Phytoplasma can now be detected using molecular biology methods [7, 8], which has allowed PaWB disease to be explored in more detail. Thus, several candidate genes that might be responsive to PaWB phytoplasma have been reported in tree species [9–11]. MicroRNAs (miRNAs) have been shown to mediate gene expression in response to biotic and abiotic stress conditions [12–17]. In plant-microorganism interactions, seven miRNAs (named miR399, miR397, miR390, miR396, miR172, miR166, and miR164) have been reported to be responsive to *Papaya meleira* virus infection [18, 19], and the target genes of miR1447, miR472, miR1448, and miR482 have been also found to encode putative disease resistance proteins in *Populus trichocarpa* [20, 21]. Futhermore, the Phytoplasma-responsive miRNAs have been reported in Mexican lime [22] and mulberry [23]. Some paulownia miRNAs have been discovered in diploid and tetraploid plants [14, 24, 25] but, to our knowledge, no miRNAs responsive to PaWB phytoplasma have been reported in the *Paulownia tomentosa* × *Paulownia fortunei*, a paulownia hybrid species. The discovery and characterization of phytoplasma-responsive miRNAs in Paulownia may provide new insights to understand the plant-phytoplasma interactions.

In plants, epigenetic modification plays an extremely important role in normal development, and provides the impetus for morphological plasticity and phenotypic diversity. In associated with epigenetic modifications, DNA methylation played the important roles in inducing gene silencing, and restarting or changing gene expression levels [14]. DNA methylation is necessary for plant development and hypomethylation or hypermethylation can influence plant traits such as yield, fruit ripening, seed size, flowering time, plant size, plant stature, sex determination, and pathogen resistance [26–30]. Indeed, in a previous study, we found that the level of DNA methylation in PaWB diseased Paulownia seedlings was lower than that in healthy seedlings, and that the DNA methylation level increased when the phytoplasma was eliminated [31]. Moreover, previous study have also demonstrated that the use of MMS to treat PaWB-infected paulownia seedlings could increase the DNA methylation level of the plants and eliminate the phytoplasma [31]. Seedlings infected by different paulownia species need to be treated with different MMS concentrations in order to eliminate the phytoplasma and recover the healthy phenotype [9, 10]. Thus, in the present study, to detect and characterize phytoplasma-responsive miRNAs in Paulownia, four small RNA (sRNA) libraries and four degradome sequencing libraries were constructed from healthy, PaWB-infected, and PaWB-infected 15 mg·L^−1^and 30 mg·L^−1^ MMS-treated plants. We discovered 33 (13 conserved and 20 novel) miRNAs belonging to 21 miRNA families that were responsive to PaWB disease. In addition, 166 targets for 10 differentially expressed conserved miRNAs and eight novel miRNAs were obtained, and their biological functions in relation to the response of Paulownia to PaWB phytoplasma infection are discussed.

## Materials and Methods

### Plant material and treatments

All plant materials were obtained from the Forestry Biotechnology Laboratory, Henan Agricultural University, Zhengzhou, Henan Province, China. *Paulownia tomentosa* × *Paulownia fortunei*, PTF (healthy), and PTFI (PaWB-infected) seedlings were cultured *in vitro* for 30 days. Then, the PTFI seedlings were transferred to triangular flasks containing1/2Murashige and Skoog (MS) culture medium [31] containing 0 (PTFI), 15 (PTFI15) or 30 mg L^−1^ MMS (PTFI30). The PTF seedlings were transferred to the triangular flasks containing 1/2 MS culture medium without MMS as the control group. After 5 days cultured at 20°C in the dark, all the samples were cultured for 30 days at 25 ± 2°C with a 16/8-h (light/dark) photoperiod. Three parallel plant samples were cultured for each condition. After culturing, nine different seedlings from each treatment group (PTF, PTFI, PTFI15 and PTFI30) were collected and mixed subsequently. The excised samples were frozen immediately, and stored at −86°C for extraction of RNA.

### Small RNA construction and sequencing

Total RNA were isolated from each mixed group using Trizol reagent (Invitrogen, Carlsbad, CA). SRNAs libraries were constructed from four groups and sequenced using HiSeq2000 system (Illumina, San Diego, CA, USA). Briefly, sRNA fragments (18–30 nt) were isolated and purified by using polyacrylamide gel electrophoresis, and then ligated to 5′ and 3′adaptors by T4 RNA ligase (Takara, Dalian, China). After reverse transcription and amplification, the products were sequenced on an Illumina HiSeq 2000 platform. The sequencing data used in this study have been submitted to the NCBI’s NIH Short Read Archive database, and accession number of SPR057548 (PRJNA281785) was assigned.

### Bioinformatics analysis and miRNA identification

After removing the low quality reads, adapters and 5' primer contaminated reads, the obtained clean reads were analyzed for their length distribution, and then mapped onto the database of Paulownia UniGenes (http://www.ncbi.nlm.nih.gov/sra) [10] using SOAP (http://soap.genomics.org.cn/). Perfectly matched reads were aligned to the Rfam and GenBank (http://www.ncbi.nlm.nih.gov/genbank/) to discard the rRNA, tRNA, snRNA, scRNA, snoRNA, and other ncoRNA sequences. The remaining reads were aligned to sequences in miRBase (Release 21.0, http://www.mirbase.org/ftp.shtml) to identify known miRNAs. Reads that aligned to known miRNAs from other plant sequences with two or fewer mismatches were regarded as potential conserved miRNAs. The novel miRNAs were screened using Mireap (https://sourceforge.net/projects/mireap/) to identify the secondary structures that are characteristic of precursor miRNAs. Subsequently, to obtained the authentic novel miRNAs, the processing precision rates for the secondary structures were calculated using the formula of Goettel *et al*. and Ma *et al*., and the miRNAs with the precision values < 0.1 were filtered [32, 33]. Other criteria described by Meyers *et al*. [34] were used to annotate the potential novel miRNAs.

### Differential expression analysis of conserved and novel miRNAs

**Fig 1:**
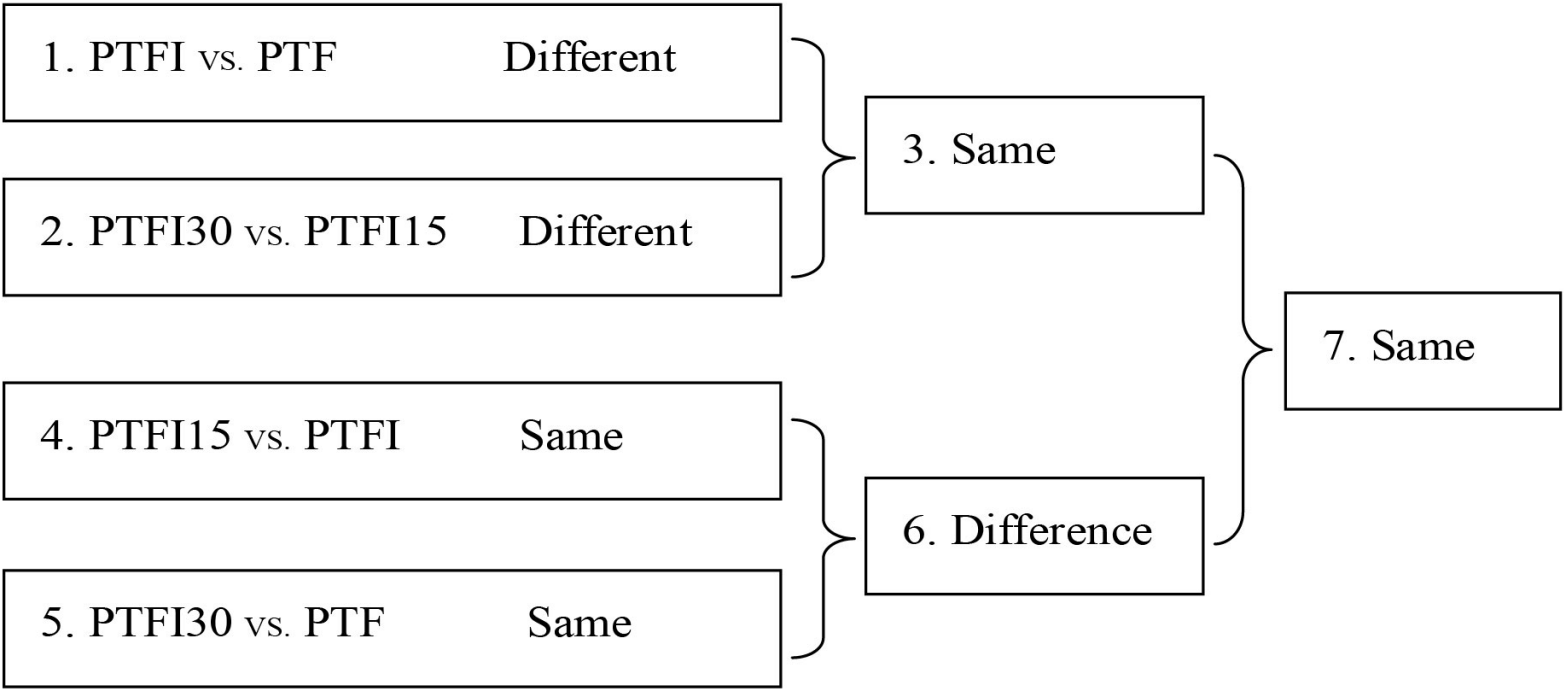
Comparison schemes of the four samples (PTF, healthy plants; PTFI, PaWB-infected plants; PTFI-15, PaWB-infected 15 mg·L−1 MMS-treated plants; PTFI-30, PaWB-infected 30 mg·L−1 MMS-treated plants).

Comparisons of miRNA expression profiles among the different samples were carried out based on seven pairwise comparisons as shown in Fig 1. First, the abundances for each miRNAs in four different libraries were normalized to get the expression of reads per million. The fold changes between any two libraries were calculated as fold change = log2 (normalized read counts in one library/normalized read counts in another library). Then statistical analysis was performed by calculating the Poisson distribution. Finally, miRNAs with fold changes ≥1.0 or ≤−1.0 and *P*-values <0.05 were considered to be significantly different between the two samples. The *P*-value was calculated as follows:

$$P\left(x|y\right)=\left(\frac{N_{2}}{N_{1}}\right)\frac{\left(x+y\right)!}{x!y!{\left(1+\frac{N_{2}}{N_{1}}\right)}^{\left(x+y+1\right)}}$$

$$C\left(y\leq y\min|x\right)=\sum_{y=0}^{y\leq y\min}p\left(y|x\right)$$

$$D\left(y\geq y\max|x\right)=\sum_{y\geq y\max}^{\infty }p\left(y|x\right)$$

### Identification of targets for Paulownia miRNAs by degradome analysis

Four degradome sequencing libraries were constructed using mRNAs isolated from the PTF, PTFI, PTFI15, and PTFI30 plants according to the protocols described previously [35] and sequenced on an Illumina HiSeq 2000 system. After low-quality sequences and adapters were removed, the obtained clean reads were aligned to the Paulownia UniGenes (http://www.ncbi.nlm.nih.gov/sra) using SOAP software. The reads with a perfect matching are used to identify miRNA-mediated cleaved fragments with CleaveLand 3.0, as previously described [35]. All the putative target genes were used as queries in BLASTX searches (E-value cutoff of 10^−5)^ against sequences in the NCBI Nr databases. The best homologs were selected and their Gene Ontology (GO) annotations (http://www.geneontology.org/) were assigned to the corresponding putative targets. In addition, a pathway analysis was performed for the targets using Blastall program (E-value threshold of <10^−5^) hits against the KEGG Pathway database (http://www.genome.jp/kegg/).

### Quantitative real-time polymerase chain reaction analysis for miRNAs and targets

The expression levels of randomly selected miRNAs and their targets were examined by quantitative real time PCR (qRT-PCR). Total RNA were extracted from the four different groups of samples, with three biological replications for each. The primers for the miRNAs and target genes were designed based on the methods described elsewhere [14, 36]. All the sequences of primers used for qRT-PCR in this study are listed in S1 Table. The qRT-PCR analysis was subjected to CFX96 real time PCR platform (Bio-Rad, Hercules, CA, USA). The PCR conditions were 50°C for 3 min, 95°C for 5 min, then 40 cycles of 95°C for 15 sec, 55°C for 30 sec, and 40°C for 10 min. All reactions were run in triplicate. The U6 and the 18S rRNA of Paulownia were chosen as the endogenous reference genes for miRNA and target mRNA normalization, respectively. The relative expression levels of the miRNAs and targets were calculated using the method of Livak and Schmittgen [37].

## Results

### Illumina sequencing of small RNAs

**Fig 2:**
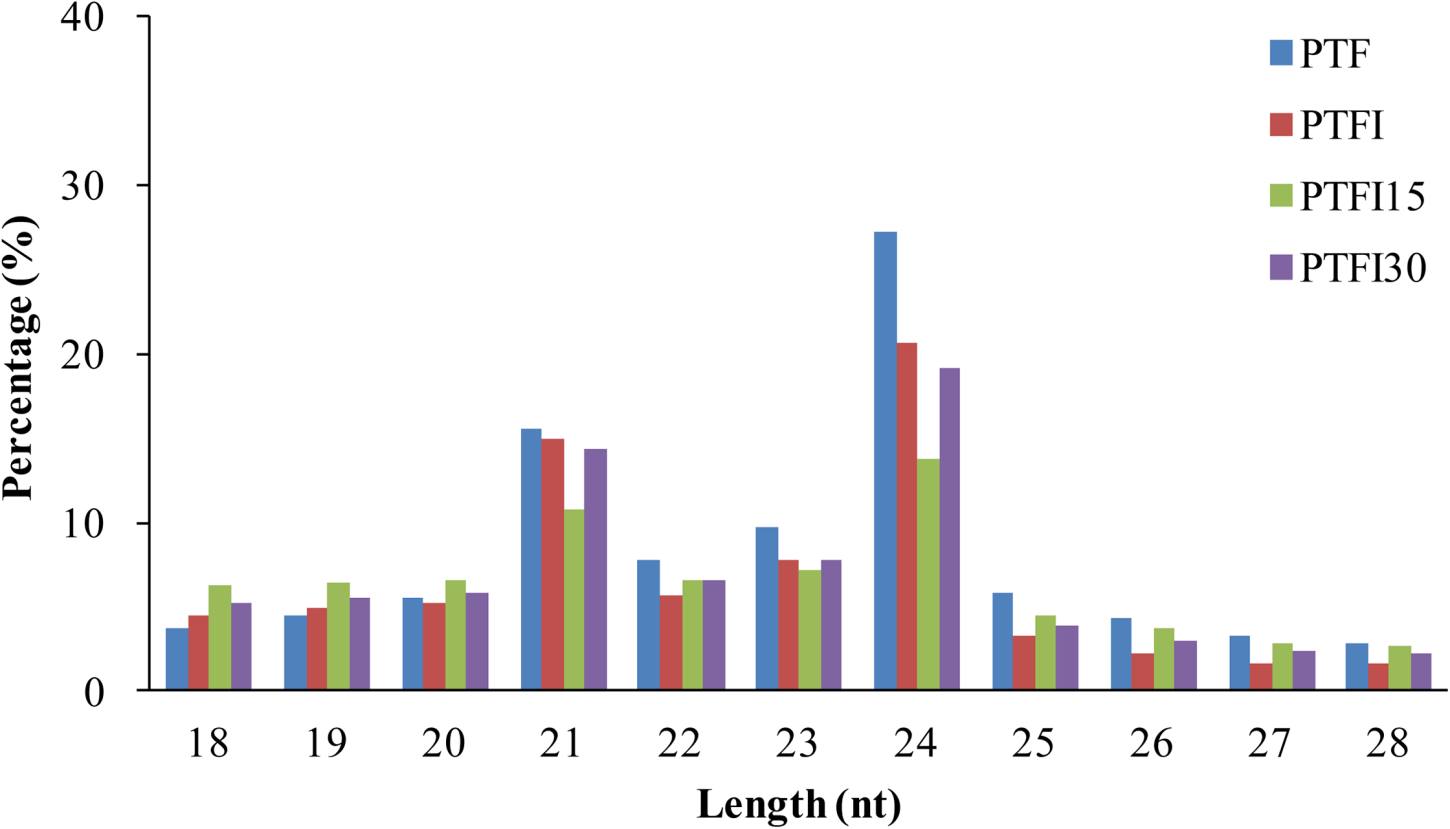
Length distribution of sRNAs in *Paulownia tomentosa × Paulownia fortunei*.

Four sRNA libraries were subjected to HiSeq 2000 sequencing. After the initial processing, 19114542 (3984607 unique), 10705047 (3022412 unique), 10283783 (1972310 unique) and 11404212 (2680769 unique) clean reads were obtained from the PTF, PTFI, PTFI15, and PTFI30 libraries, respectively. The clean reads included miRNA, rRNA, snRNA, snoRNA, and tRNA sequences, and other unannotated reads (Table 1). Most of the reads in all four libraries were 20–24 nt in length (Fig 2), which is consistent with the size from Dicer-like digestion products and is also reported on previously published results [14, 24]. The 24-nt sRNAs were the most abundant, with approximately 27.17%, 20.69%, 13.76%, and 19.07%, present in the PTF, PTFI, PTFI15 and PTFI30 libraries, respectively, followed by the 21-nt sRNAs, which suggested that the 21- and 24-nt sRNAs may play important roles in PaWB disease resistance in Paulownia.

**Table 1:** Annotation of sRNAs sequences in four libraries.

category		total	miRNA	rRNA	snRNA	snoRNA	tRNA	unannote
**PTF**	**Unique sRNAs**	3984607	37390	95957	3600	2591	14676	3830393
	**Percent%**	100%	0.94%	2.41%	0.09%	0.07%	0.37%	96.13%
	**Total sRNAs**	19114542	2474294	4631875	21954	36931	328108	11621380
	**Percent%**	100%	12.94%	24.23%	0.11%	0.19%	1.72%	60.80%
**PTFI**	**Unique sRNAs**	3022412	32787	75848	2812	2044	11882	2897039
	**Percent%**	100%	1.08%	2.51%	0.09%	0.07%	0.39%	95.85%
	**Total sRNAs**	10705047	1619222	2196299	11085	24992	207313	6646136
	**Percent%**	100%	15.13%	20.52%	0.10%	0.23%	1.94%	62.08%
**PTFI15**	**Unique sRNAs**	1972310	21371	91730	3695	2637	11322	1841555
	**Percent%**	100%	1.08%	4.65%	0.19%	0.13%	0.57%	93.37%
	**Total sRNAs**	10283783	970671	3682200	18233	39566	224412	5348701
	**Percent%**	100%	9.44%	35.81%	0.18%	0.38%	2.18%	52.10%
**PTFI30**	**Unique sRNAs**	2680769	28820	80528	3005	2145	11638	2554633
	**Percent%**	100%	1.07%	3.00%	0.11%	0.08%	0.43%	95.29%
	**Total sRNAs**	11404212	1536500	2909013	14780	29611	203381	6710927
	**Percent%**	100%	13.47%	25.51%	0.13%	0.26%	1.78%	58.85%

### Identification of conserved miRNAs

To identify conserved miRNAs, the sRNA sequences that remained after removing the rRNA, scRNA, snoRNA, snRNA, tRNA, and other ncoRNA sequences were queried against the sequences in miRBase (http://www.mirbase.org/). We identified 125 conserved miRNAs (118 for PTF, 125 for PTFI, 117 for PTFI15, and 119 for PTFI30) belonging to 26 families (S2 Table). Of these, 94 complementary miRNA* sequences were also identified in the four libraries, which has been considered as evidence of authentic miRNAs. The average precursor length of the conserved miRNAs was 143 nt and ranged from 75 to 305 nt. The minimal folding free energy (MFE) of the precursor sequences varied from −34.50 to −109.10 kcal/mol, with an average energy of −55.70 kcal/mol (S2 Table).

The miR159, miR166, and miR397 families were represented most frequently in the four libraries; the miR482, miR398, miR397, miR396, andmiR2118 families were moderately abundant in the four libraries, while the miR395 and miR530 families had no more than 100 copies and miR477 was absent from the PTF library. Thus, some differences in the miRNA populations in the four libraries were detected. We also found that the abundances of the miRNAs in nine miRNA families (miR4414, miR397, miR396, miR395, miR394, miR169, miR168, miR167, and miR156) were the same in the PTF and PTFI60 libraries, and the number of reads of miRNA394 and miRNA395 in the PTFI library has equivalent to that in the PTFI15 library. Moreover, the abundance of MiR4414 in the PTFI15 library was greater than in the PTFI library. The abundances of members of the other miRNA families were fewer in PTFI15 library compared with their abundances in the PTFI library.

### Identification of novel miRNAs

Mireap software was employed to detect potentially novel miRNAs from the unannotated sRNAs in the four libraries. The processing precision values were calculated for the secondary structures and shown in S3 Table. As a result, 92, 93, 76, and 79 candidate novel miRNAs (processing precision value > 0.1) were obtained in the PTF, PTFI, PTFI15, and PTFI30 libraries, respectively (S3 and S4 Tables). After removing redundant sequences, 118 novel miRNAs were identified in the four libraries, the lengths of the miRNA sequences with the majority being 22-nt long. The MFEs of the candidate novel miRNAs varied from −19.10 to −152.20 kcal/mol in the four libraries, with average MFE values of −54.03 kcal/mol in PTF, −52.31 kcal/mol in PTFI, −52.15 kcal/mol in PTFI15, and −53.18 kcal/mol in PTFI30. Most of these identified novel miRNAs exhibited at low detection frequencies; and only 46 of them were expressed in all four libraries.

### MiRNAs associated with the PaWB disease response in Paulownia

The four samples were evaluated in seven pairwise comparisons (Fig 1) and miRNAs that significantly differentially expressed in each comparison were identified (S2 and S4 Tables). The expression levels of 113 (28 conserved and 85 novel) and 132 (64 conserved and 68 novel) miRNAs were significantly different in the PTFI vs. PTF (1) and PTFI30 vs. PTFI15 (2) comparisons, respectively. Among these miRNAs, 64 (16 conserved and 48 novel) that were consistently differentially expressed in the two comparisons were retrieved (3). In the PTFI15 vs. PTFI (4) and PTFI30 vs. PTF (5) comparisons, 72 (35 conserved and 37 novel) and 176 miRNAs (110 conserved and 66 novel) were identified, respectively. Moreover, 154 miRNAs (78 conserved and 76novel) were detected in the pairwise comparison (6). Finally, 33 (13 conserved and 20 novel) miRNAs belonging to the 21 families were detected in comparison (7).

### Target genes detected by degradome sequencing

Degradome sequencing identified 814 target genes for 23 conserved miRNA families and 32 novel miRNAs. There were 591 targets in the PTF library, 313 (53.0%), 13 (2.2%), 141 (23.9%), 8 (1.4%), and 116 (19.6%) of which were grouped into categories 0, I, II, III, and IV according to the previous study [35], respectively (S1 Fig and S5 Table). In the PTFI library, 664 targets were identified and 330 (49.7%), 24 (3.6%), 109 (16.4%), 23 (3.5%), and 178 (26.8%) of them were grouped into categories 0, I, II, III, and category IV, respectively. Of the 606 targets in the PTFI15 library, 277 (45.7%), 21 (3.5%), 200 (33.0%), 8 (1.3%), and 100 (16.5%) were grouped into categories 0, I, II, III, and IV, respectively. Of the 601 targets in the PTFI30 library, 281 (46.8%), 12 (2.0%), 210 (34.9%), 6 (1.0%), and 92 (15.3%) were grouped into categories 0, I, II, III, and IV, respectively. Out of the 814 target genes, 166 targets were predicted to be cleaved by 10 differentially expressed conserved miRNAs (pau-miR168a/b/c/d/e, pau-miR169h/i, pau-miR4414a/b, and pau-miR530) and eight novel miRNAs (pau-mir32, pau-mir34, pau-mir90a,b, pau-mir41 and pau-mir46a,b,c) that were considered to be potential PaWB disease-related miRNAs (S5 Table). Based on their biological functions predicted after mapping them to the Nr, GO, and KEGG Pathway databases, some of the 166 target genes may be involved in plant biotic and abiotic stress resistance (S2 Fig and S5 Table). For example, the targets annotated as serine/threonine-protein phosphatase BSL3-like, protein kinase PVPK-1-like isoform 1, nbs-lrr resistance protein, putative late blight resistance protein homolog R1A-10-like, and benzoate carboxyl methyltransferase were targeted by pau-mir32, pau-mir34, and pau-mir41. These results suggested that target genes that are responsive to PaWB disease have been detected in this study.

### Validation of candidate PaWB disease-responsive genes by qRT-PCR

**Fig 3:**
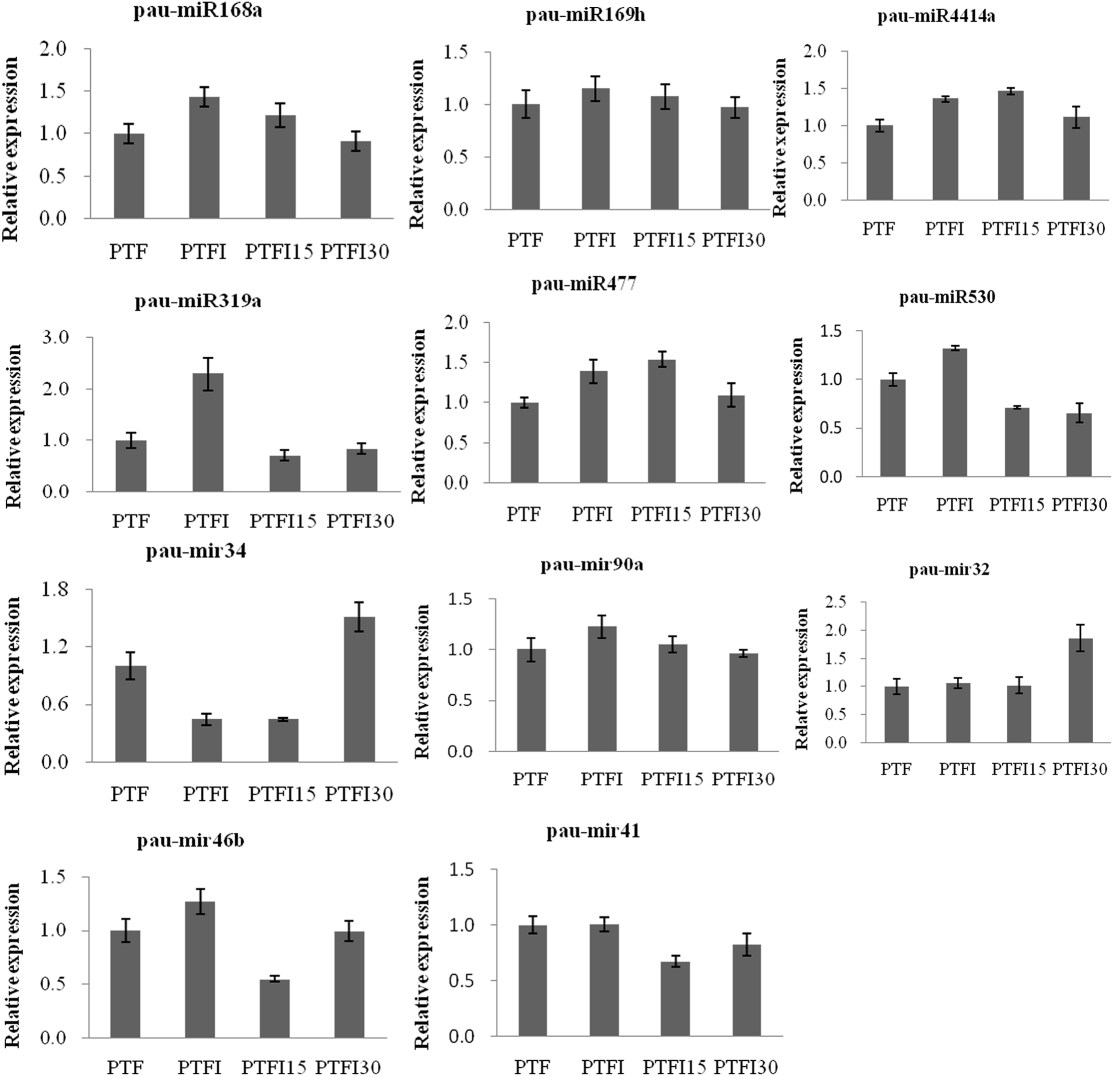
Quantitative RT-PCR analysis of miRNAs in *Paulownia tomentosa × Paulownia fortunei*. PTF, healthy plants; PTFI, PaWB-infected plants; PTFI-15, PaWB-infected 15 mg·L−1 MMS-treated plants; PTFI-30, PaWB-infected 30 mg·L−1 MMS-treated plants.Three biological replicates for each plant samples were performed. Expression values are means ± SD (n = 3). U6 was used as the internal reference gene, and the normalized miRNA levels in the PTF were arbitrarily set to 1.

**Fig 4:**
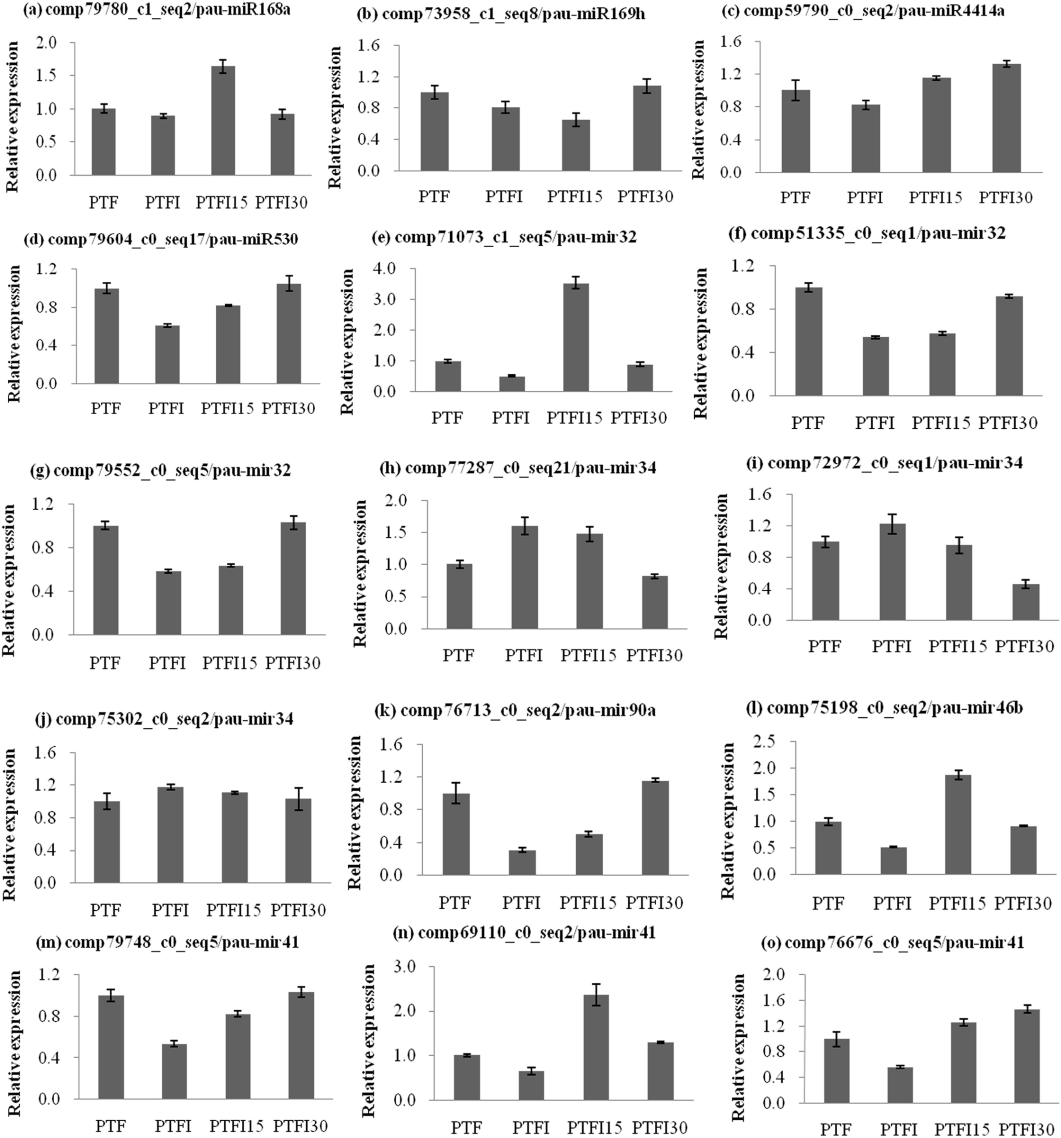
Validation of target gene expression levels in *Paulownia tomentosa × Paulownia fortunei*. PTF, healthy plants; PTFI, PaWB-infected plants; PTFI-15, PaWB-infected 15 mg·L−1 MMS-treated plants; PTFI-30, PaWB-infected 30 mg·L−1 MMS-treated plants. (a) target expression levels for a argonaute1-1. (b) target expression levels for anuclear transcription factor Y. (c) target expression levels for a subtilase. (d) target expression levels for a uncharacterized protein. (e) target expression levels for a ADP-ribosylation factor 1-like. (f) target expression levels for a brassinosteroid insensitive 1-associated receptor kinase 1. (g) target expression levels for a serine/threonine-protein phosphatase. (h) target expression levels for a nbs-lrr resistance protein. (i) target expression levels for a auxin-induced protein PCNT115-like. (j) targetexpression levels for a lectin-receptor like protein kinase 3. (k) target expression levels for a pentatricopeptiderepeat-containing protein. (l) target expression levels for a uncharacterized protein. (m) target expression levels for a calcium-transporting ATPase 4. (n) target expression levels for a glutathione S-transferase T1-like. (o) target expression levels for a Elongation factor TuA. Three biological replicates for each plant samples were performed. Expression values are means ± SD (n = 3). 18SrRNA was used as the internal reference gene, and the normalized target levels in the PTF were arbitrarily set to 1.

To validate the results obtained from the Illumina sequencing data, 11 miRNAs and 15 target genes from the four libraries were subjected to qRT-PCR assays. The results showed that there was strong correlation between the read abundances in the sequencing data and the expression levels obtained by qRT-PCR, indicating that the miRNA expression profiles estimated from the Illumina sequencing data were quantitative and reliable (Fig 3). The correlation between the expressions of the target genes and their corresponding miRNAs was also analyzed. The results showed that the expression levels of ten miRNA-target gene pairs were negatively correlated (Figs 3 and 4), except for pau-mir32, pau-miR168a, and pau-mir41 and their targets serine/threonine-protein phosphatase (comp79552_c0_seq5), ADP-ribosylation factor 1-like (comp71073_c0_seq2), brassinosteroid insensitive 1-associated receptor kinase 1 (comp51335_c0_seq1), argonaute1-1 (comp79780_c1_seq2), and Elongation factor TuA (comp76676_c0_seq5), which displayed a positive and inconsistent correlation, respectively (Figs 3 and 4).

## Discussion

### Small RNAs in the Paulownia libraries

In recent years, Illumina platform has provided us a large number of conserved miRNAs and novel miRNAs in model organisms such as rice and Arabidopsis as well as in non-model organisms. In this study, to understand the molecular mechanisms that operate between the PaWB disease-causing phytoplasma and its host, we characterized the sRNAs in four Paulownia libraries based on the Paulownia UniGene transcriptome information. Here, more than 10million clean reads were obtained from the PTF, PTFI, PTFI15 and PTFI30 libraries, respectively. The total percentage of 21–24 ntsRNAs was more than 90% in all the libraries, which is consistent with previously published results in the other Paulownia species, and suggested that the sequencing data from our sRNA libraries were reliable [14, 24].

### Expression patterns of Paulownia miRNAs under the phytoplasma stress

Hundreds of Paulownia miRNAs have been identified [14, 24, 25], but not much is known about the miRNAs that may be involved in the plant’s response to the PaWB phytoplasma. In this present study, we obtained 125 conserved miRNAs belonging to 26 families and 118 novel miRNA from four sRNA libraries constructed from the leaves of four treatments (healthy, PaWB-infected, PaWB-infected and 15 mg L^−1^ MMS-treated, and PaWB-infected and 30 mg L^−1^ MMS-treated plants). Compared with previous work on the phytoplasma miRNAs from two sRNA libraries (healthy and infected plants) in the Mexican lime [20] and mulberry [21], the present study identified the PaWB phytoplasma responsive miRNAs in the *P*. *tomentosa*× *P*. *fortunei* and the dynamic expression patterns of miRNAs under the pathogen-induced stress and different MMS concentration treatments.

The expressions of some of the identified miRNAs exhibited decreased abundance in infected and symptomatic plants (PTFI and PTFI15), and then increased abundance in the PTFI60 library, including pau-miR156, pau-miR159a/b/c/d, pau-miR166g/h/i/j, pau-miR482e/f, pau-miR397a/b/c/d, pau-miR396c/d, pau-mir9, pau-mir50a/b, pau-mir61, and pau-mir62 (S2 and S4 Tables); the expressions of some miRNAs demonstrated the opposite trends, that is increased abundance in infected and symptomatic plants (PTFI and PTFI15) and decreased abundance in the PTFI60 library, including pau-miR169e, pau-miR319e/f/g, pau-miR4414a/b, pau-miR477, and pau-mir90a/b (S2 and S4 Tables); and the expressions of some of them displayed the irregular trends. Moreover, we also found 13 miRNAs (pau-miR169a/f/g, pau-miR171d, pau-miR319c/d, pau-mir90a/b, pau-mir71, pau-mir75, pau-mir86a/b, and pau-mir92) that were present only in the symptomatic PTF and PTFI15 plants, and 13 miRNAs (pau-mir16, pau-mir21, pau-mir34, pau-mir48a/b, pau-mir61,and pau-mir62) that were present only in the non-symptomatic PTF and PTFI30 plants (S2 and S4 Tables). These findings indicated that the relation between the differentially expressed miRNAs and the PaWB phytoplasma was rather complicated.

Furthermore, the expressions of miRNAs and their target genes were also tested by the qRT-PCR experiment (Figs 3 and 4). The result showed that the expression of ten target genes have the inverse correlation with the expression of their corresponding miRNAs (Figs 3 and 4). Among them, two target genes comp73958_c1_saq8 and comp59790_c0_seq2 that encode the nuclear transcription factor Y and subtilase were expressed at relatively lower levels in infected and symptomatic plants (PTFI and PTFI15) than in PTF and PTFI30 plants. The corresponding miRNAs pau-miR169h and pau-miR4414a, respectively, were expressed in the inverse pattern. In contrast, the genes comp77287_c0_seq21 (nbs-lrr resistance protein), comp72972_c0_seq1 (auxin-induced protein PCNT115-like) and comp75302_c0_seq2 (lectin-receptor like protein kinase 3) have the relatively higher expression levels in the PTFI and PTFI15, whereas the expression of pau-mir34 in the infected and symptomatic plants showed the lower trend than that in PTF and PTFI30. Moreover, the expressions of pau-miR530 and pau-mir90a were inversely correlated with the expression of the target gene, uncharacterized protein (comp79604_c0_seq17) and pentatricopeptide repeat-containing protein (comp76713_c0_seq2), which were decreased in the PTFI plant and then increased in the PTFI15 and PTFI30 samples. This negative correlation between the miRNAs and the target gens were also reported in the other plant species, such as wheat, cotton, Arabidopsis, maize, and *Paulownia australis* [14, 15, 38–40]. Interestingly, the expressions of some of miRNAs and their targets have not showed the perfectly negative correlations in our results. For example, compared with the expression levels in the PTF, PTFI15 and PTFI30 plants, pau-miR168a and pau-mir41 showed higher levels in the PTFI plant, respectively; while the reverse patterns were true for their targets encoding the argonaute1-1 (comp79780_c1_seq2), calcium-transporting ATPase 4 (comp79748_c0_seq5), and elongation factor TuA (comp76676_c0_seq5). However, the inconsistencies between the levels of miRNAs (pau-miR168a and pau-mir41) and their targets have been found in the PTF, PTFI15 and PTFI30 plants. These results are in consistence with the former verified results that the other correlation between the expressions of miRNAs and their target genes was existed [38, 41]. In the present study, the expression levels of the miRNAs and their target genes did not show a perfectly negative correlation and did not show a regular tend with the amounts of PaWB phytoplasma in the host. This absence of a perfect negative correlation is likely because one miRNA can have multiple target genes, and these genes can interact with each other and influence the expression patterns of the miRNAs [41–43]. Another reason for the irregular trend may be attributed to temporal expression among the miRNAs.

### Targets for phytoplasma-responsive miRNAs in Paulownia

Degradome sequencing identified 814 potential targets for the 23 conserved miRNA families and 32 novel miRNAs. According to the biological function annotations, we found that our results were quite similar to previous reports that a large number of the targets in Paulownia encode transcription factors, including SPLs, ARFs, MYBs, NACs, GRFs, and nuclear transcription factor Y (NF-Y) [12, 14, 39, 40]. We also noted that 166 genes were targeted by the 10 conserved miRNA families and eight novel miRNAs that were considered previously to be PaWB disease-related miRNAs in Paulownia (S2, S4 and S5 Tables). These target genes were involved mainly in metabolic pathways including plant-pathogen interaction and plant hormone signal transduction. For instance, the degradome sequencing analysis showed that the differentially expressed miRNAs pau-miR169h,i targeted nine Paulownia transcripts that encode NF-Y subunit A (NF-YA), also called CCAAT-specific binding factor, which is known to be involved in response to stresses in other plants [44, 45]. Inal et al. has reported that miR169 family numbers play the important roles in the wheat plant under the *Fusarium culmorum* and *Bipolaris sorokiniana* stresses by negatively regulated their target genes encoding for the Pto kinase 1 [15]. Moreover, the expression levels of miR169 family numbers were detected to be upregulated in the poplar [46], while the expressions were downregulated in soybean and wheat cultivars in response to fungal stress [15, 47]. In addition, the expression of six miR169 family numbers were significantly induced in Paulownia against the PaWB phytoplasma stress, and the expression levels of rest numbers were increased slightly. Similarly, the expression of miR395b was downregulated in the two wheat cultivars (-2.19 and -1.19 fold) under the *Fusarium culmorum* pathogen attack [15], but slightly upregulated in the Paulownia (0.43 fold) in response to phytoplasma stress. These findings indicated that there exist some different tolerance mechanisms among the difference host plants under the biotic stresses and that the patterns of miRNA expressions showed a dynamic changing process under the pathogen induced stress. AGO1 is the main component of miRNA biogenesis, and is involved in regulating diverse physiological processes such as, cellular, developmental, signaling, immune system, negative or positive regulation of biological process, and response to stimulus [48–50]. In a study of the *Verticillium longisporum* induced *Brassica napus*, the expression levels of miR168 and its target AGO1 were altered in the infected plants compare with the control [51]. Moreover, Ellendorff et al. [52] has suggested the *Arabidopsis thaliana* AGO1 mutants showed enhanced resistance and greatly reduced disease symptoms compared with the wild type. We found that the miR168 family numbers were differentially expressed in PaWB-stressed Paulownia, suggesting that miR168 might act as an important regulator in a compatible Paulownia–phytoplasma interaction.

Interestingly, we also found novel miRNAs that play important roles in plant tolerance or response to stresses. Among them, DEAD-box ATP-dependent RNA helicase 22-like and ras-related protein RABF1 were targeted by the differentially expressed miRNA, pau-mir32. DEAD-box RNA helicases form the largest family of RNA helicases possessing the conserved sequence DEAD in motif II, and its crucial roles in plant growth and developmental regulation has been validated by Linder and Jankowsky in the previous study [53]. Ras proteins, also called small GTPases, generally serve as molecular switches in various cellular signaling events, have been reported to regulate the three stages (initiation, elongation, and termination) of protein biosynthesis, and play an important role in cell proliferation and differentiation [54]. These findings suggested that pau-mir32 may be involved in the symptomatic appearance of the specific proliferation symptoms in PaWB-diseased Paulownia plants. Furthermore, we have also identified four genes that encode BAK1 (brassinosteroid insensitive 1-associated receptor kinase 1) and three genes that encode EF-Tu (translation elongation factor Tu). It has reported that the BAK1may be involved in cell death control and brassinosteroid signaling in Arabidopsis; and the conserved domain in EF-Tu could trigger pathogen-associated molecular pattern (PAMP)-triggered immunity, which has been tested and proven in *Brassicaceae* [55, 56]. PAMPs are essential components in the complex defense network in a plant’s response to fight off invading pathogens [57, 58]. In this study, genes targeted by the novel miRNAs pau-mir32, pau-mir34, and pau-mir41, were annotated as BAK1, and EF-Tu, respectively, indicating that these miRNAs and their targets may be involved in plant hormone signal transduction and plant-pathogen interaction pathways. In addition to the targets mentioned above, the differentially expressed miRNAs pau-mir32, pau-mir41, pau-mir90a,b and pau-mir34 targeted other plant-pathogen-related genes encoding the serine/threonine-protein phosphatase BSL3-like, protein kinase PVPK-1-like isoform 1, pentatricopeptide repeat-containing protein, nbs-lrr resistance protein, NBS type disease resistance protein, putative late blight resistance protein homolog R1B-12-like (R1B-12), R1B-16, R1B-17, and R1A-10. According to the GO analysis, these target genes are associated to the biological processes that biological regulation, cellular process, metabolic process, regulation of biological process, response to stimulus, signaling, single-organism process, binding, and catalytic activity. Thus, our results supported the hypothesis that pau-mir32, pau-mir41, pau-mir90a,b and pau-mir34 have the important effect on helping enhance the Paulownia tolerance to stresses caused by phytoplasma. Further studies are required to understand the biological functions of the remaining differentially expressed miRNAs for which no targets were found. The molecular mechanism regulated by these miRNA-target interactions may be involved in the processes that enhance plant tolerance or adaptation to the PaWB pathogen.

## Conclusions

In this study, we investigated systematically miRNAs and their targets in the healthy and diseased *P*. *tomentosa*× *P*. *fortunei* at the transcriptome-wide level by Illumina sequencing and a degradome approach, and provided a useful resource for further investigations of paulownia-phytoplasma interactions. We detected 125 conserved and 118 novel miRNAs in four libraries and discovered 33 differentially expressed miRNAs in Paulownia that were responsive to the PaWB disease. The target genes for these differentially expressed miRNAs were involved mainly in plant-pathogen interactions and plant hormone signal transduction metabolic pathways. These findings will provide new directions for further experimental studies of the host response to phytoplasma infection in Paulownia and other plant species. Due the different genetic backgrounds among the paulownia species, to thoroughly understand the molecular mechanisms that underlying the PaWB disease and its host, further investigation of phytoplasma-responsive miRNAs in the other paulownia species treated with the suitable MMS concentrations is required.

## Supporting Information

S1 Fig The categories of target plots (t-plots) inidentified miRNA targets by degradome sequencing. (TIF)

S2 Fig Gene Ontology analysis of PaWB disease-related miRNA target genes in *Paulownia tomentosa × Paulownia fortunei*. (TIF)

S1 Table TheqRT-PCR used primers of selected miRNAs and their targets. (XLSX)

S2 Table The conserved miRNA identified in the *Paulownia tomentosa × Paulownia fortunei*. (XLSX)

S3 Table The processing precision values for the identified novel miRNAs. (TXT)

S4 Table Novel miRNAs identified from *Paulownia tomentosa × Paulownia fortunei*. (XLSX)

S5 Table Identification of miRNA targets in *Paulownia tomentosa × Paulownia fortunei* by degradome analysis. (XLSX)
