# Supplementary figures and images for: Plant–Pathogen Interaction-Related MicroRNAs and Their Targets Provide Indicators of Phytoplasma Infection in Paulownia tomentosa × Paulownia fortunei
Source: PLoS One. 2015 Oct 20;10(10):e0140590. doi: 10.1371/journal.pone.0140590 (PMC4617444; doi:10.1371/journal.pone.0140590)

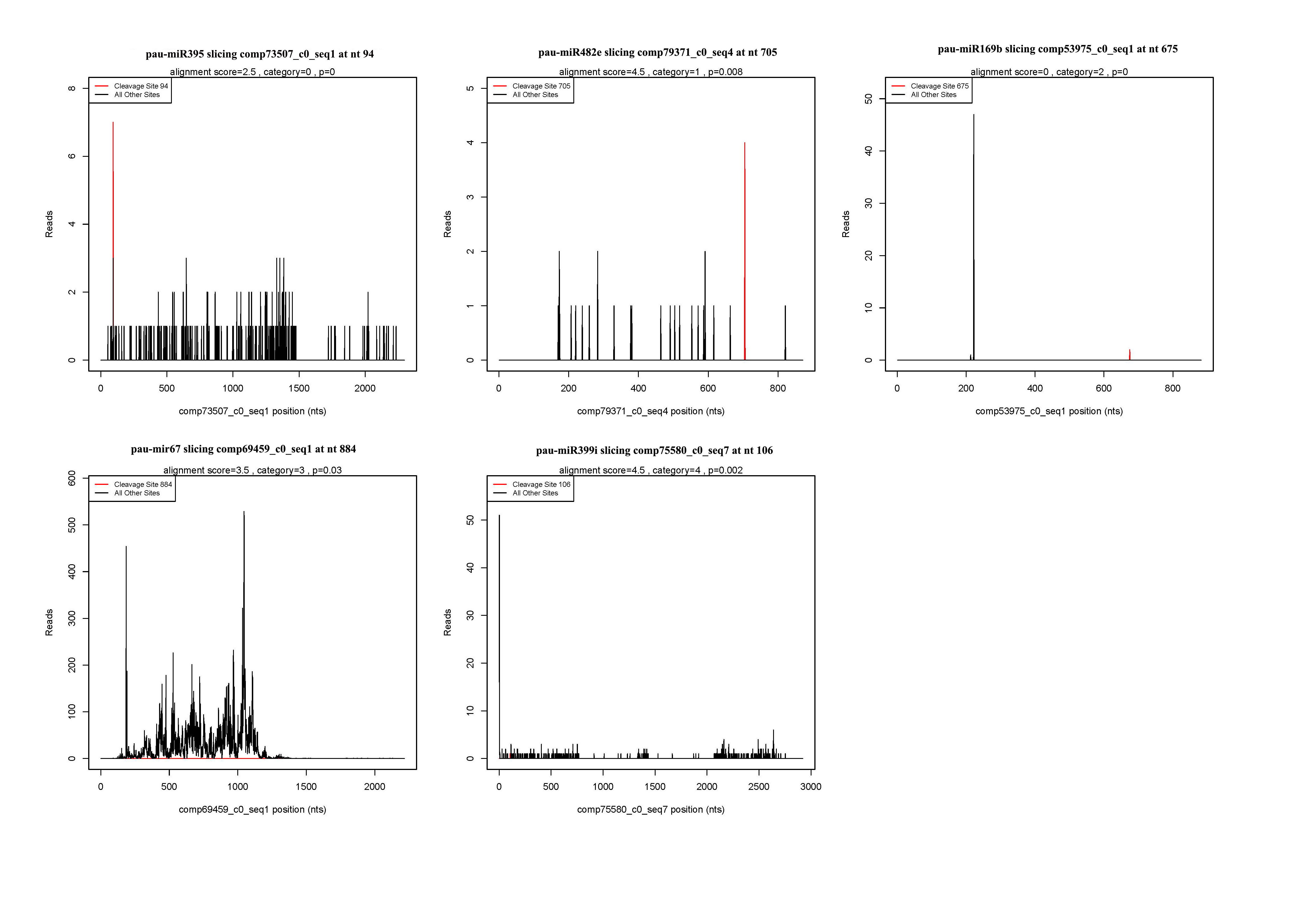

Supplement: S1 Fig — (TIF) [file pone.0140590.s001.tif]

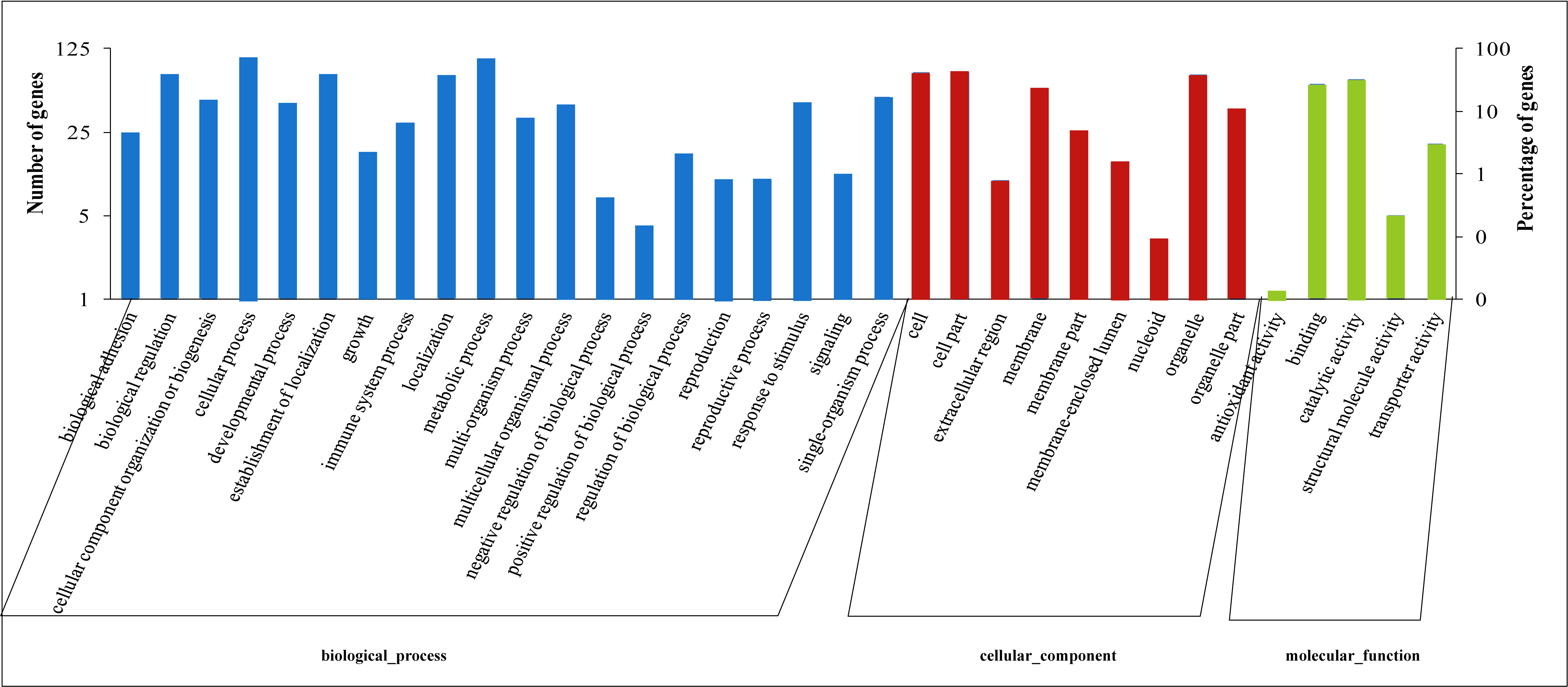

Supplement: S2 Fig — (TIF) [file pone.0140590.s002.tif]
